# Predicting poor neurological outcomes following out-of-hospital cardiac arrest using neuron-specific enolase and neurofilament light chain in patients with and without haemolysis

**DOI:** 10.1093/ehjopen/oead078

**Published:** 2023-08-28

**Authors:** Yusuf Abdi Isse, Ruth Frikke-Schmidt, Sebastian Wiberg, Johannes Grand, Laust E R Obling, Anna Sina Pettersson Meyer, Jesper Kjaergaard, Christian Hassager, Martin A S Meyer

**Affiliations:** Department of Cardiology, The Heart Center, Rigshospitalet, Copenhagen University Hospital, Blegdamsvej 9, DK2100 Copenhagen, Denmark; Department of Clinical Biochemistry, Center of Diagnostic Investigation, Copenhagen University Hospital Rigshospitalet, Copenhagen, Denmark; Department of Clinical Medicine, University of Copenhagen, Copenhagen, Denmark; Department of Cardiology, The Heart Center, Rigshospitalet, Copenhagen University Hospital, Blegdamsvej 9, DK2100 Copenhagen, Denmark; Department of Cardiology, The Heart Center, Rigshospitalet, Copenhagen University Hospital, Blegdamsvej 9, DK2100 Copenhagen, Denmark; Department of Cardiology, The Heart Center, Rigshospitalet, Copenhagen University Hospital, Blegdamsvej 9, DK2100 Copenhagen, Denmark; Department of Cardiology, The Heart Center, Rigshospitalet, Copenhagen University Hospital, Blegdamsvej 9, DK2100 Copenhagen, Denmark; Department of Cardiology, The Heart Center, Rigshospitalet, Copenhagen University Hospital, Blegdamsvej 9, DK2100 Copenhagen, Denmark; Department of Clinical Medicine, University of Copenhagen, Copenhagen, Denmark; Department of Cardiology, The Heart Center, Rigshospitalet, Copenhagen University Hospital, Blegdamsvej 9, DK2100 Copenhagen, Denmark; Department of Clinical Medicine, University of Copenhagen, Copenhagen, Denmark; Department of Cardiology, The Heart Center, Rigshospitalet, Copenhagen University Hospital, Blegdamsvej 9, DK2100 Copenhagen, Denmark

**Keywords:** Neuron-specific enolase, Neurofilament light chain, Out-of-hospital cardiac arrest, MoCA, Haemolysis, Free-haemoglobin

## Abstract

**Aims:**

Hypoxic-ischaemic brain injury following out-of-hospital cardiac arrest (OHCA) is a common complication and a major cause of death. Neuron-specific enolase (NSE) and neurofilament light chain (NfL) are released after brain injury and elevated concentrations of both are associated with poor neurological outcome. We explored the influence of haemolysis on the prognostic performance of NSE and NfL.

**Methods and results:**

The study is based on *post hoc* analyses of a randomized, single-centre, double-blinded, controlled trial (IMICA), where comatose OHCA patients of presumed cardiac cause were included. Free-haemoglobin was measured at admission to quantify haemolysis. NSE and NfL were measured after 48 h to estimate the extent of brain injury. Montreal Cognitive Assessment score (MoCA) was assessed to evaluate neurocognitive impairments. Seventy-three patients were included and divided into two groups by the median free-haemoglobin at admission. No group differences in mortality or poor neurological outcome were observed. The high-admission free-haemoglobin group had a significantly higher concentration of NSE compared to the low-admission free-haemoglobin group (27.4 µmol/L vs. 19.6 µmol/L, *P* = 0.03), but no differences in NfL. The performance of NSE and NfL in predicting poor neurological outcome were high for both, but NfL was numerically higher [area under the ROC (AUROC) 0.90 vs. 0.96, *P* = 0.09]. Furthermore, NfL, but not NSE, was inversely correlated with MoCA score, *R*^2^ = 0.21, *P* = 0.006.

**Conclusion:**

High free-haemoglobin at admission was associated with higher NSE concentration after 48 h, but, the performance of NSE and NfL in predicting poor neurological outcome among OHCA patients were good regardless of early haemolysis. Only elevated NfL concentrations were associated with cognitive impairments.

## Graphical Abstract

**Graphical abstract:**
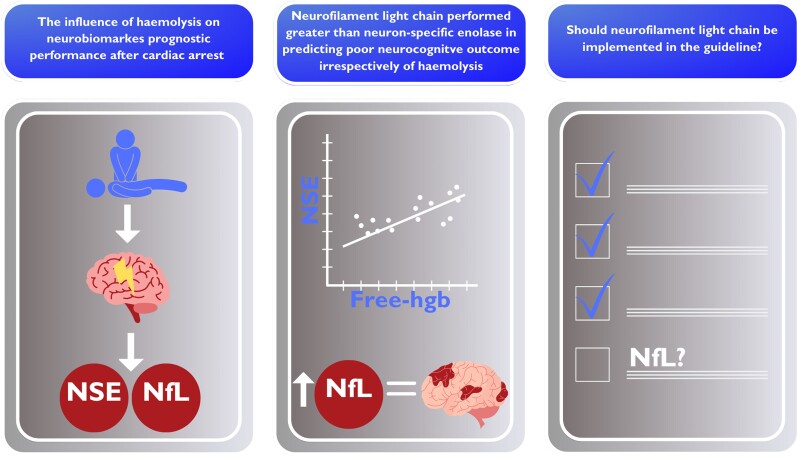

## Introduction

Individuals resuscitated from out-of-hospital cardiac arrest (OHCA) and admitted to hospital in coma have <50% chance of survival to discharge.^1,2^ After successful resuscitation, patients are at high risk of developing post-cardiac arrest syndrome as a complication including hypoxic-ischaemic brain injury.^3–6^ In the early phase after OHCA, cardiovascular failure is the most common cause of death, while withdrawal of life-sustaining treatment (WLST) due to presumed severe hypoxic-ischaemic brain injury is accountable for most of the later deaths.^1,7^ Furthermore, long-term cognitive impairments have been reported in 40–50% of OHCA survivors and guidelines recommend early cognitive screening and referral to specialist rehabilitation when signs of cognitive impairments are identified.^8^

Current guidelines suggest a multimodal approach for neurological prognostication including clinical examination, electroencephalography, imaging, and measuring neuron-specific enolase (NSE) as a neurological biomarker.^8^ NSE is a glycolytic intracellular enzyme located in neuroectodermal-derived cells, including neurons.^9^ Several studies have observed a correlation between high NSE concentrations and poor neurological outcome among OHCA patients.^10^ However, the same biomarker has been detected in other cells including red blood cells and haemolysis may potentially interfere with the interpretation.^9^ The guidelines recommend measuring haemolysis index (free-haemoglobin) in each sample and discard samples with an exceed index level.^8^ Samples with a haemolysis index at the time of measuring within a prespecified interval will get adjusted according to standard laboratory practice. However, the adjustment does not account for early haemolysis during or after cardiac arrest, which is a common phenomenon.^11^ This is due to the very short half-life of free-haemoglobin (free-hgb) in contrast to NSE with a considerably longer half-life.^12^ Potentially, NSE might be elevated at 48 h due to release from destructed red blood cells during resuscitation.^13^ Therefore, it is essential to investigate if early haemolysis has an impact on the interpretation of NSE. Yet, no studies have routinely measured early haemolysis among OHCA patients and determined its impact on later NSE measurements.

Neurofilament light chain (NfL) is an emerging neurobiomarker found in the cytoskeleton of neurons. NfL has shown a better prognostic accuracy in detecting patients with poor neurological outcome at 24 h and 48 h after OHCA compared to NSE.^14^ Furthermore, the localization of NfL in other cell lines beside neurons has not been reported, which makes this biomarker specific for neuronal damage.

Our aim was to investigate the influence of haemolysis upon admission on the performance of NSE and NfL in predicting poor neurological outcome.

## Methods

All patients included in this study participated in the IMICA trial (March 2019 to December 2019); a single-centre, randomized clinical trial, in which 80 comatose OHCA patients of presumed cardiac aetiology were randomized to receive either a single 1-h infusion of tocilizumab of 8 mg/kg (maximum 800 mg) or placebo in addition to standard care. This substudy is an explorative analysis of serum biomarkers from the IMICA trial.^15,16^ Inclusion criteria were adult age (≥18 years), OHCA with presumed cardiac cause, unconsciousness (Glasgow Coma Scale <9), and sustained return of spontaneous circulation (ROSC). For exclusion criteria see published protocol.^16^

The primary objective of the IMICA trial was to investigate the efficacy of tocilizumab on reducing high sensitivity C-reactive protein among comatose OHCA patients while the secondary objectives included determining the effect of tocilizumab on reducing organ injury, and improving neurological outcome characterized by cerebral performance category (CPC) as well as survival at 180 days. Initially, written informed consent was obtained from relatives according to national legislation. Informed consent from participants were afterward obtained when the participants gained consciousness. This study was approved by the regional ethics committee of The Capital Region of Denmark (Approval No.: H-18037286) and the Danish Medicines Agency (Approval No.:2018-002686-19). The IMICA trial was registered in www.clinicaltrials.gov (Identification No.: NCT03863015).^14^

### Biomarker measurements

Routine blood samples from arterial lines were collected at 0 and 48 h after admittance. The blood samples for routine biochemistry and biobank were investigated locally at the Department of Clinical Biochemistry at Rigshospitalet. Photometry was used to measure free-hgb. The lower level of detection for free-hgb was 3 µmol/L, and samples with a concentration below 3 µmol/L were reported as 3 µmol/L. NSE was measured for clinical use by COBAS 8000 e801 module using an electro-chemi-luminescent-immuno-assay (ECLIA) kit (Roche Diagnostica, Switzerland). The measuring range of NSE was set at 0.2 µg/L to 300 µg/L and a clinical decision concentration at 16.3 µg/L. In each NSE sample, the haemolysis index was systematically measured for quality assurance according to standard laboratory practice. An increased haemolysis index between 10 and 80 mg/dL was corrected using this formula: NSE (µg/L)_corrected_ = NSE (µg/L)_measured_—0.184×[haemolysis index (mg/dL)]. Samples with a haemolysis index below 10 mg/dL were not adjusted, and samples with a haemolysis index above 80 mg/dL were discarded, and a new blood sample was collected immediately. Blood samples were collected and prepared for storage by spinning whole blood at 2000g for 10 min at—80°C. We performed a *post hoc* analysis of biobank serum samples to assess NfL utilizing the enzyme-linked immunosorbent assay (ELISA) method with an R-PLEX Human Neurofilament L Antibody assay in a MESO QuickPlex SQ120 (MSD, Rockville, Maryland).

### Assessment of functional and cognitive performance

The CPC (range 1–5) was performed 180 days after admission to evaluate greater neurological disability. Poor neurological outcome was defined as a CPC ≥3. To evaluate cognitive impairments the Montreal Cognitive Assessment score (MoCA) (range 0–30) was assessed at 90 days follow up and a MoCA score of 0–10 was defined as severe cognitive impairment, 11–22 as moderate cognitive impairment, 23–25 as mild cognitive impairment, whereas a score of 26 or above was considered normal cognitive function.^17,18^ Each clinical score was performed by a healthcare professional.

### Statistical analysis

Continuous variables are presented as median and 25th–75th percentiles and analysed by non-parametric methods. Categorical variables are presented as numbers and percentages and analysed by Fischer’s exact test or χ^2^ test for between-group comparisons. To predict poor neurological outcome, logistic regression and receiver operating characteristics curve (ROC) with area under the ROC (AUROC) were produced. Regression analysis (SAS proc reg.) was performed to investigate the association between NSE, NfL, and free-hgb, as well as MoCA and neurobiomarkers after log2-transformation. Kruskal–Wallis one-way analysis of variance was used to determine differences across MoCA classification groups for NSE and NfL. A *P-*value of <0.05 was considered statistically significant. SAS Enterprise Guide 7.1 (SAS-Institute Inc) was used to perform statistical analysis.

## Results

A total number of 80 patients were included in this study; of these seven patients did not have free-hgb measured at admission and were therefore excluded from further analysis. The main trial found no differences in mortality or poor neurological outcome between the intervention group (tocilizumab) and control group (placebo). Furthermore, the interventions in the main study did not influence the concentrations of NSE (*P* = 0.22), NfL (*P* = 0.92), or free-hgb at admission (tocilizumab 10.0 µmol/L [4-24] vs. placebo 15.0 µmol/L [5-22], *P* = 0.44). Thirty patients (37.5%) had a poor neurological outcome (CPC 3–5) at 180 days and the mortality was 28 (35.0%) in this study. A total of 35 out of 46 survivors had a MoCA test available at follow-up, with no difference in scores between the tocilizumab and control group (*P* = 0.72), and the median MoCA score was 27 (25–29). For further analysis, the sample size is divided into two groups by the median free-hgb concentration measured at admission 14 µmol/L (5.0–22.0): high admission free-hgb group and lowadmission free-hgb group. The patients in the high admission free-hgb group had a significantly longer time to ROSC compared to the low admission free-hgb group (*Table 1*), but mortality, poor neurological outcome (CPC 3–5), and cognitive impairments assessed by MoCA score did not differ between the groups. Furthermore, the high admission free-hgb group did not differ from the low admission free-hgb group with respect to age, sex, bystander cardiopulmonary resuscitation (CPR), or first monitored rhythm being shockable.

**Table 1:** Baseline characteristics and outcome

Characteristics				
	All	Low admission free-haemoglobin group	High admission free-haemoglobin group	*P*-value
Patients (*n*)	80	36	37	
Age, median (IQR) (y)	62.5 (54.0–71.0)	66 (57.0–72.0)	61 (54.0–70.0)	0.26
Sex, male (no %)	66 (82.5)	27 (75.0)	32 (86.5)	0.25
Time to ROSC median (IQR) (min)	20.0 (13.0–27.0)	14.0 (10.0–20.0)	25.0 (19.0–40.0)	<0.01*
Bystander CPR, yes (no %)	67 (83.8)	27 (75.0)	34 (91.9)	0.06
First monitored rhythm, shockable (no %)	74 (92.5)	31 (88.6)	36 (97.3)	0.19
Free-hgb at admission, median (IQR) (µmol/L)	14.0 (5.0–22.0)	4.5 (3.0–8.5)	22.0 (17.0–36.0)	<0.01*
*Outcome*				
Free-hgb 48 h, median (IQR) (µmol/L)	3.0 (3.0–3.0)	3.0 (3.0–3.0)	3.0 (3.0–3.0)	0.27
NSE 48 h, median (IQR) (µg/L)	21.6 (16.9–42.9)	19.6 (14.9–24.2)	27.4 (18.4–126.0)	0.03*
NfL 48 h, median (IQR) (pg/L)	84.5 (36.1–860.4)	80.4 (35.0–213.7)	94.5 (36.4–3039.8)	0.51
CPC at 180 days, 3–5 (No %)	30 (37.5)	12 (33.3)	16 (43.2)	0.47
Mortality at 180 days, (No %)	28 (35.0)	11 (30.6)	16 (43.2)	0.33
Moca score at, median (IQR) (No)	27 (25–29)	27 (24–28)	28 (25–29)	0.48

* indicates *p* < 0.05.

Abbreviations: CPC, Cerebral Performance Category scale; CPR, cardiopulmonary resuscitation; hgb, haemoglobin; IQR, interquartile range; MoCA, Montreal Cognitive Assessment score NfL, neurofilament light chain; NSE, neuron-specific enolase; ROSC, return of spontaneous circulation.

### Prognostic performance of NSE and NFL

**Figure 1:**
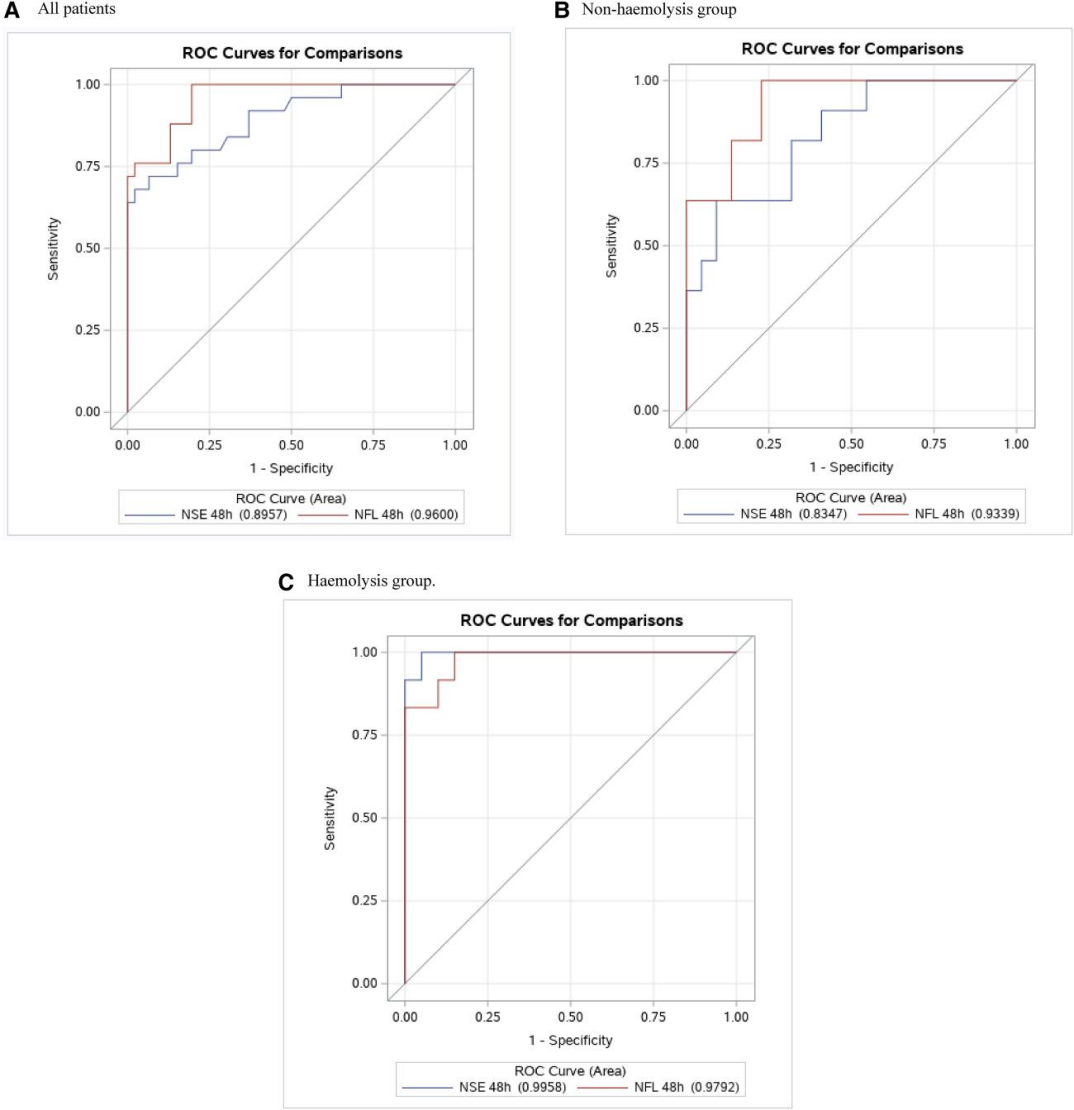
Prognostic performance of neuron-specific enolase and neurofilament light chain in predicting poor neurological outcome after out-of-hospital cardiac arrest. Receiver operating characteristic plot showing the predictive ability of neuron-specific enolase and neurofilament light chain measured at 48 h after admission to predict poor neurological outcome (Cerebral performance category ≥3) at 180 days after out-of-hospital cardiac arrest in comatose patients. The sample size is divided into two groups: a non-haemolysis group (< median haemolysis) and a haemolysis group (≥ median haemolysis). (*A*) All patients. (*B*) Non-haemolysis group. (*C*) Haemolysis group.

To predict poor neurological outcome characterized by CPC at 48 h, the AUROC for NSE was 0.90 (0.82–1.00), and AUROC for NfL was 0.96 (0.92–1.00) with a difference in AUROC of 0.06 (0.00–0.14), *P* = 0.09 (*Figure 1*). Free-hgb achieved an AUROC 0.59 (0.45–0.73) and 0.50 (0.41–0.60) at 0 and 48 h, respectively. There was a strong association between NfL and NSE at 48 h, *R*^2^ = 0.65, *P* < 0.01. The association between NfL and NSE at 48 h was not significantly affected by admission free-hgb when a model combining NSE and admission free-hgb for predicting NfL was constructed (*P* = 0.13 for admission free-hgb; overall *R*^2^ = 0.66 for model). Admission levels of free-hgb was very weakly associated with the 48 h concentration of NSE (*R*^2^ = 0.12, *P* < 0.01; see Supplementary material online Figure S1) but not NfL (*R*^2^ = 0.03, *P* = 0.16). To predict cognitive impairment, NfL was inversely associated with MoCA score, *R*^2^ = 0.21, *P* < 0.01 (see Supplementary material online, *Figure S2*). In contrast, no significant association between NSE measured at 48 h and MoCA score was observed, *R*^2^ = 0.02, *P* = 0.40. Further, NfL concentrations (*P* <0.01), but not NSE (*P* = 0.69), varied according to the classification of no, mild, or moderate cognitive impairments assessed by MoCA score (no survivors had a MoCA score classified as severe) (see Supplementary material online, *Figure S3*).

### High admission free-haemoglobin group vs. Low admission free-haemoglobin group

Free-hgb at 48 h was 3.0 µmol/L (3.0–3.0) in both groups. Overall, the concentration of NSE at 48 h was 21.6 µmol/L (16.9–42.9) and the high admission free-hgb group had a significantly higher concentration of NSE compared with the low admission free-hgb group (27.4 µmol/L vs. 19.6 µmol/L, *P* = 0.03). For NfL the total concentration was 84.5 pg/mL (36.1–860.4) at 48 h and no significant difference in NfL concentrations was seen when comparing the high admission free-hgb group to the low admission free-hgb group (94.5 pg/L vs. 80.4 pg/L, *P* = 0.51). Furthermore, the performance of NSE and NfL in predicting poor neurological outcomes was similar when comparing the high admission free-hgb group and low admission free-hgb group at 48 h (*Figure 1*). Even when examining the performance of NSE and NfL in the patients with the highest quartile of admission free-hgb (>22 µmol/L) the performance of both remained unaffected with no sign of a difference between AUROC for NSE and NfL (AUROC 1.00 and 0.96, respectively; *P* = 0.32 for a difference).

## Discussion

In confirmation of previous studies,^14,19^ NSE and NfL had an overall strong discriminatory ability to predict poor neurological outcome after OHCA, with NfL demonstrating a numerically greater predictive accuracy in comparison to NSE. This observation aligns with the findings of a large meta-analysis investigating neurological outcome after cardiac arrest utilizing neurobiomarkers for prognostication.^20^ When stratifying patients based on higher or lower admission free-hgb, we found that patients with high free-hgb after OHCA had longer time to ROSC and higher NSE concentrations after 48 h compared to the group with low free-hgb, however, no group difference in NfL was seen. Wihersaari *et al.* conducted a *post hoc* analysis of a randomized clinical trial investigating the prognostic performance of NfL among comatose OHCA patients, and the study found a positive correlation between haemolysis and NSE concentration, however, NfL measurements were independent of haemolysis.^14^ Our results parallel these findings, although our samples are separated in time, as haemolysis was measured at admission and markes our brain injury were measured at 48 h, and the associatione between admission free-hgb and NSE was very weak. Also, the predictive performance of NSE and NfL in the present study was similar when comparing high admission free-hgb group and low admission free-hgb group. In terms of predicting cognitive performance, NfL showed a descriptive ability to stratify by severity in cognitive impairment and was inversely associated with the MoCA score, which was not observed for NSE. A large biobank study has investigated the association between NfL and four different assessment methods evaluating long-term neurocognitive outcome among OHCA survivors, and found that serum NfL demonstrated a significant association with all four methods from small to moderate association in which CPC had the strongest correlation 0.41 (95% CI: 0.32–0.49).^21^ This might suggest that measurement of NfL in the early phase of admittance may identify patients at risk of developing cognitive impairment. We encourage future studies to investigate NfL in a larger clinical setting and to determine its ability to predict cognitive dysfunction.The release of NfL reflects axonal injury to neurons and the majority of the white matter consists of axons. Other modalities such as magnetic resonance imaging (MRI) are used to visualize structural damages including white matter lesions. A study by Velly *et al.* utilized diffusion MRI to quantify white matter injury after OHCA and showed a promising prognostic performance for long-term neurological outcome assessed by CPC with an AUROC of 0.95 (CI: 0.91–0.98).^22^ A combined approach using both biomarkers and imaging that visualizes white matter might better determine patients’ ultimate neurocognitive outcome.

Two clinical studies have investigated the correlation between neurological biomarkers and haemolysis among patient undergoing elective open-heart surgery with extracorporeal circulation. Both studies found proportionality between NSE and free-hgb *r* = 0.56^12^ and *r* = 0.77,^23^ respectively, and the strongest correlation was seen 2 h after surgery, while no correlation between NfL and free-hgb was found. Moreover, several *in vitro* studies have investigated the correlation between induced haemolysis in blood samples and NSE concentrations, and a strong correlation has been found (*R*^2^ > 0.95).^9,24^ This indicates that haemolysis during sample collection has a stronger interference with NSE measurements compared to haemolysis induced by cardiac arrest or elective open-heart surgery. Therefore, the investigator must be aware of haemolysis during sample collection and routinely measure the haemolysis index. Currently, NSE values are only adjusted among samples with a high haemolysis index at the corresponding timepoint, however, early haemolysis associated with cardiac arrest is not taken into account, which may affect the serum concentration of NSE.^13^ Our results show that patients with a high admission free-hgb had a higher NSE at 48 h, but a higher mortality rate or an increased incidence of poor neurological outcome were not observed in this group. This might indicate that the slightly increased NSE concentration was driven by destructed red blood cells during OHCA, which can be explained by the longer time to ROSC. However, NfL concentrations measured at 48 h were not influenced by the degree of haemolysis at admission, and further, the association between NfL and NSE at 48 h was not significantly affected by admission free-hgb.

The two clinical studies that have been conducted in patients undergoing heart surgery, examined the immediate effects of haemolysis during surgical procedure on neurobiomarkers,^12,23^ and similarly the previous study in cardiac arrest patients examined the influence of haemolysis occouring at the time of sampling for the neurobiomarkers.^14^ Our study differs from these clinical trials because we investigated the influence of haemolysis upon admission on the performance of NSE and NfL measured 48 h after admission, since these values at this timepoint have an important prognostic role on the decision-making for continuing life-sustaining treatment.

This study has some general limitations. Primarily, our results are based on a small sample size and it is a secondary analysis of a randomized clinical trial, and therefore, must be considered descriptive. Furthermore, NSE was measured in a clinical setting with no freeze-thaw cycle while NfL samples were frozen and stored in biobank and afterwards thawed for analysis. Second, our findings are applicable to measurement of NSE and NfL 48 h after admission and may not apply to NSE and NfL measured at later timepoints. Finally, these results apply to OHCA patients of presumed cardiac cause and might not be applied with certainty to noncardiac causes, or to more extreme ranges of haemolysis.

## Conclusion

In the present study, we found that patients with high free-haemoglobin at admission had a higher NSE concentration at 48 h compared to the group with low admission free-haemoglobin. Both NSE and NfL measured at 48 h after admission had a strong prognostic capability for poor neurological outcome among comatose OHCA patients independent of early haemolysis. These findings indicate that early haemolysis does not affect the predictive ability of NSE in identifying patients with a high risk of a poor neurological outcome in a general OHCA population with a cardiac ethology. Additionally, elevated NfL concentrations at 48 h were associated with cognitive impairment assessed by MoCA score at 3 months follow up.

## Supplementary Material

oead078_Supplementary_Data

## Data Availability

The supporting data of this study are available from the corresponding author upon reasonable request and after approvals from relevant authorities.

## Lead author biography

Yusuf Abdi Isse earned his medical degree from the University of Copenhagen in Denmark and currently working as a research assistant at ‘Critical cardiac care research group’ at the Heart Center, Rigshospitalet, Denmark. His research focus includes out-of-hospital cardiac arrest, neuroprognostication, and preventing post-cardiac arrest syndrome.
