# Supplementary material for: Predicting poor neurological outcomes following out-of-hospital cardiac arrest using neuron-specific enolase and neurofilament light chain in patients with and without haemolysis
Source: Eur Heart J Open. 2023 Aug 28;3(4):oead078. doi: 10.1093/ehjopen/oead078 (PMC10461601; doi:10.1093/ehjopen/oead078)
Supplement: oead078_Supplementary_Data [file oead078_supplementary_data.docx]

# Supplementary Appendix

Predicting poor neurological outcomes following out-of-hospital cardiac arrest using neuron-specific enolase and neurofilament light chain in patients with and without hemolysis.

Contents

[Supplementary Appendix 1](#_Toc143158380)

[**Supplementary figure S1:** The association between neurobiomarkes and free-hemoglobin. 2](#_Toc143158381)

[**Supplementary figure S2:** Scatter plot of Montreal Cognitive Assessment score at 3 month follow up and Neurofilament light chain measured at 48 hours. 4](#_Toc143158382)

[**Supplementary figure S3:** Neurobiomarkers according to Montreal Cognitive Assessment score. 5](#_Toc143158383)

## **Supplementary figure S1:** The association between neurobiomarkes and free-hemoglobin.

A)

**
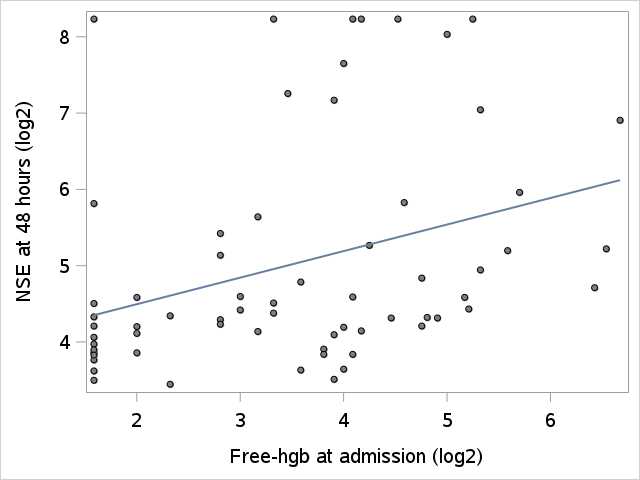
**

**B)
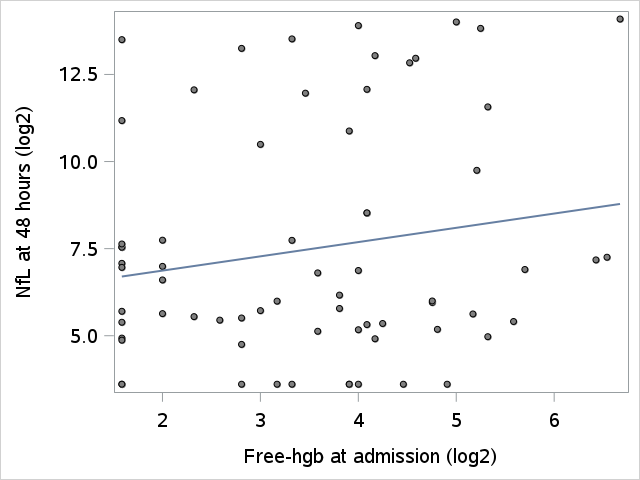
**

Figure legend: Scatter plot of the association between neurobiomarkes measured 48 hours after hospital admission and admission free-hemoglobin. Both variables are log2 transformed. A) Neuron-specific enolase (NSE) [µg/L] and admission free-hemoglobin. B) neurofilament light chain (NfL) [pg/L] and admission free-hemoglobin

## **Supplementary figure S2:** Scatter plot of Montreal Cognitive Assessment score at 3 month follow up and Neurofilament light chain measured at 48 hours.


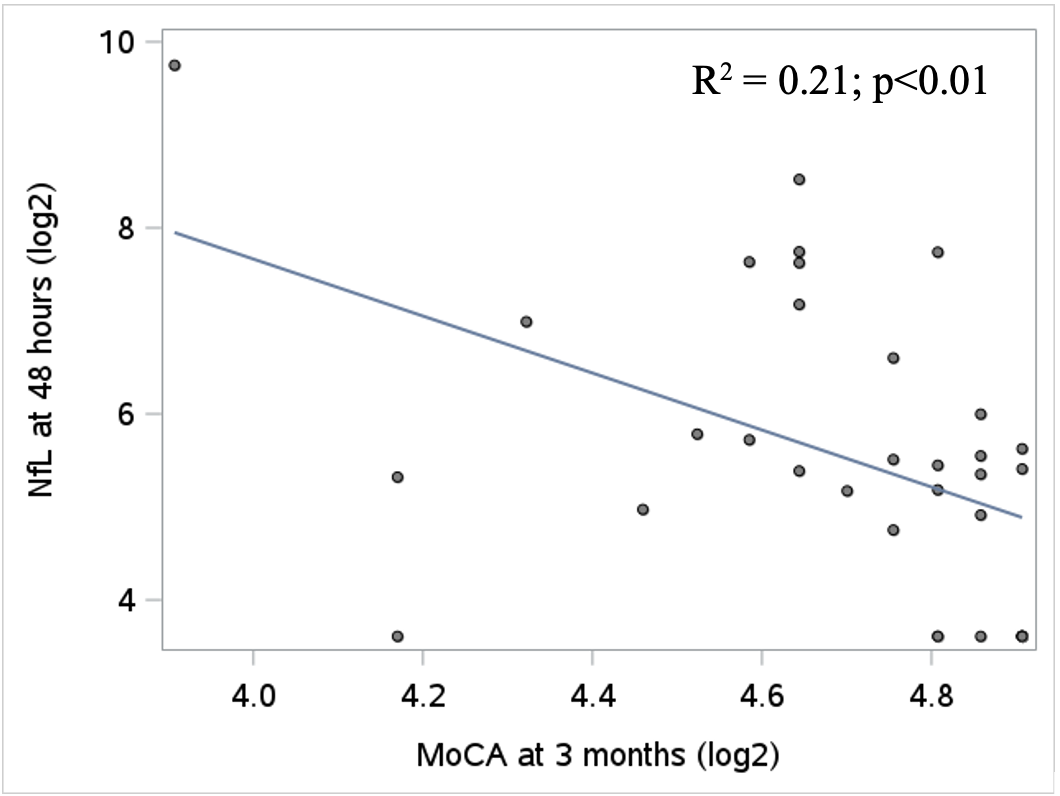


Figure legend: Scatter plot of Montreal Cognitive Assessment score at 3 month follow up and Neurofilament light chain [pg/L] measured 48 hours after hospital admission. Both variables are log2 transformed.

## **Supplementary figure S3:** Neurobiomarkers according to Montreal Cognitive Assessment score.

A)
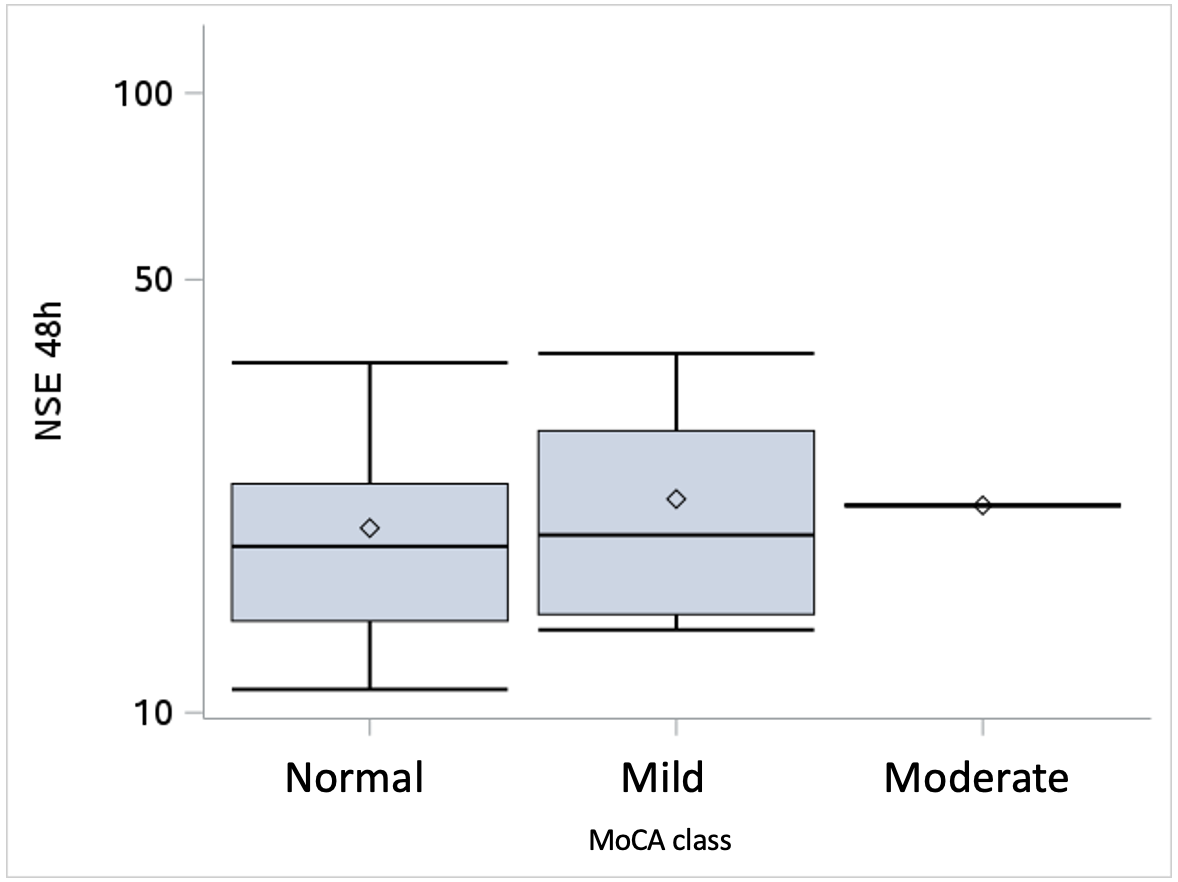


B)
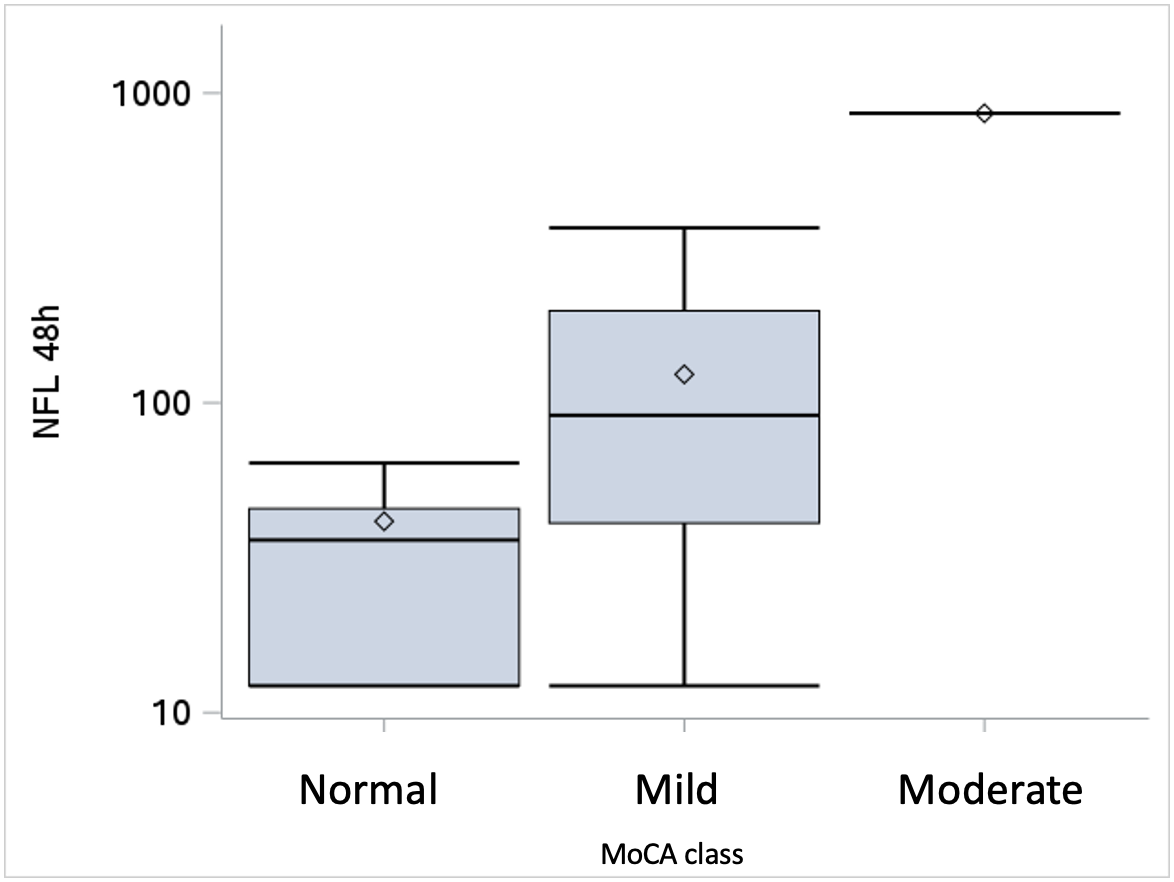


Figure legend: Neurobiomarker concentrations measured 48 hours after hospital admission according to Montreal Cognitive Assessment score (MoCA) at 3 months stratified by normal, mild, or moderate cognitive impairment. A) Neuron-specific enolase (NSE) on a logarithmic scale according to MoCA. B) Neurofilament light chain (NfL) on a logarithmic scale according to MoCA.
